# c-Src Regulates Akt Signaling in Response to Ghrelin via β-Arrestin Signaling-Independent and -Dependent Mechanisms

**DOI:** 10.1371/journal.pone.0004686

**Published:** 2009-03-05

**Authors:** Maria Lodeiro, Marily Theodoropoulou, Maria Pardo, Felipe F. Casanueva, Jesus P. Camiña

**Affiliations:** 1 Laboratory of Molecular and Cellular Endocrinology, Instituto de Investigaciones Sanitarias, Complejo Hospitalario Universitario de Santiago (CHUS), Santiago de Compostela, Spain; 2 CIBER Fisiopatología de la Obesidad y Nutrición, Santiago de Compostela, Spain; 3 Department of Endocrinology, Max Planck Institute of Psychiatry, Munich, Germany; 4 Department of Medicine, University of Santiago de Compostela, Santiago de Compostela, Spain; University of Hong Kong, Hong Kong

## Abstract

The aim of the present study was to identify the signaling mechanisms to ghrelin-stimulated activation of the serine/threonine kinase Akt. In human embryonic kidney 293 (HEK293) cells transfected with GHS-R1a, ghrelin leads to the activation of Akt through the interplay of distinct signaling mechanisms: an early G_i/o_ protein-dependent pathway and a late pathway mediated by β-arrestins. The starting point is the G_i/o_-protein dependent PI3K activation that leads to the membrane recruitment of Akt, which is phosphorylated at Y by c-Src with the subsequent phosphorylation at A-loop (T308) and HM (S473) by PDK1 and mTORC2, respectively. Once the receptor is activated, a second signaling pathway is mediated by β-arrestins 1 and 2, involving the recruitment of at least β-arrestins, c-Src and Akt. This β-arrestin-scaffolded complex leads to full activation of Akt through PDK1 and mTORC2, which are not associated to the complex. In agreement with these results, assays performed in 3T3-L1 preadipocyte cells indicate that β-arrestins and c-Src are implicated in the activation of Akt in response to ghrelin through the GHS-R1a. In summary this work reveals that c-Src is crucially involved in the ghrelin-mediated Akt activation. Furthermore, the results support the view that β-arrestins act as both scaffolding proteins and signal transducers on Akt activation.

## Introduction

The growth hormone secretagogue receptor type 1a (GHS-R1a^1^) transduces the main signals carried by ghrelin participating in the regulation of growth hormone release, food intake and energy homeostasis [1]. Ghrelin stimulation of GHS-R1a results in activation of heterotrimeric G proteins followed by receptor phosphorylation that leads to the β-arrestin recruitment, which then sterically interdicts further coupling to G proteins and targets the receptor for internalization [2], [3]. The ghrelin/GHS-R1a complex is internalized by a clathrin-mediated mechanism through an endosomal trafficking pathway. Once the ligand-receptor complex is internalized into vesicles, GHS-R1a is sorted into endosomes to be slowly recycled back to the plasma membrane [2]. This slow recycling might be determined by the stability of the complex GHS-R1a/β-arrestin during clathrin-mediated endocytosis since this complex appears to dictate the profile of the receptor resensitization [4]. Indeed, ghrelin-stimulated GHS-R1a shows significant endosomal β-arrestin recruitment [3]. In addition to the role of β-arrestin in terminating G protein signaling, recent studies have demonstrated that β-arrestins also function as scaffold molecules for numerous signaling networks such as protein kinases, including the mitogen activated protein kinases, extracellular signal-regulated kinase (ERK), c-Jun NH2-terminal Kinase (JNK), and p38 mitogen-activated protein kinase (p38), as well as Akt, PI3K and RhoA [5].

The signaling mechanisms that underlie the activation of the mitogenic ERK growth response by the GHS-R1a are complex and result from both classical G protein-regulated effectors and β-arrestin dependent ERK recruitment [6]. One pathway is G_i/o_-dependent and it is mediated by phosphatidylinositol 3-kinase (PI3K), protein kinase Cε (PKCε) and non-receptor tyrosine kinase c-Src (c-Src). The second pathway is G_q/11_-dependent and involves the activation of protein kinase Cα/β (PKCα/β) and c-Src. A third pathway involves the recruitment of GHS-R1a, c-Src, Raf-1 and ERK 1/2 into a β-arrestin-scaffolded complex. G protein and β-arrestin mediated ERK pathways are both temporally different and act in a sequential way. The β-arrestin-scaffolded signaling complex persists for prolonged periods and it is probably determined by the stability of the β-arrestin /ghrelin receptor complexes. It has become increasingly recognized that these scaffolding complexes can determine the subcellular location and specificity, promoting phosphorylation of diverse cytosolic substrates and thereby having different physiological consequences [5].

The GHS-R1a has been implicated in Akt signaling, a serine/threonine kinase that exerts a central role in the regulation of metabolism, apoptosis, transcription and cell-cycle [7]. Although previous studies have shown the activation of Akt in endothelial [8]–[11], skeletal muscle [12], thyroid [13], preadipocytes [14] and pancreatic cells [15], the molecular events that follow GHS-R1a ligand binding to trigger Akt activation remain unknown. To better understand these molecular mechanisms, we used HEK 293 cells stably expressing the GHS-R1a (HEK-GHSR1a) and 3T3-L1 preadipocyte cells. The experiments were designed to assess the roles, if any, played by G proteins and β-arrestins.

## Methods

### Materials

Human ghrelin was obtained from Global Peptide (CO, USA). Pertussis toxin was from Sigma (St. Louis, Mo, USA). Wortmannin, PP2 and PP3 were purchased from Calbiochem (San Diego, CA, USA). Anti-p44/42 MAPK rabbit polyclonal, anti-pSrc (Y416) rabbit polyclonal, anti-pAkt HM (S473), anti-pAkt A-loop (T308), anti-Akt rabbit polyclonal, anti-Rictor rabbit polyclonal, anti-mTOR rabbit polyclonal and anti-pPDK1 (S241) rabbit polyclonal antibodies were from Cell Signaling Technology (Beverly, MA, USA). Rictor siRNA and siRNA control were synthesized by Cell Signaling Technology (Beverly, MA, USA). Anti-pY rabbit polyclonal antibody was from Upstate Technology (Lake Placed, NY, USA). Anti-β-arrestin 1 goat polyclonal, anti-β-arrestin 2 mouse monoclonal and anti-c-Src rabbit polyclonal antibodies were from Santa Cruz Biotechnology (Santa Cruz, CA, USA). β-arrestin 1 siRNA, β-arrestin 2 siRNA, c-Src siRNA and siRNA control were likewise from Santa Cruz Biotechnology (Santa Cruz, CA, USA). Anti-rabbit horseradish peroxidase was from GE-Amersham, (Buckinghamshire, UK), while anti-goat horseradish peroxidase was from Santa Cruz (Santa Cruz, CA, USA). Rabbit anti-rat β-arrestin 1C-terminal (A1CT) antiserum was provided by Prof. R.J. Lefkowitz (Duke University Medical Center, Durham, NC, USA).

### Cell culture

HEK 293 cells, which stably express the human ghrelin receptor 1a (HEK-GHSR1a), were cultured as previously described [2]. 3T3-L1 preadipocyte cells were obtained from American Type Culture Collection and maintained in DMEM containing 10% calf serum, 100 U/ml penicillin, and 100 U/ml streptomycin. Cells were grown under a humidified atmosphere of 95% air, and 5% CO_2_ at 37°C.

### Cell transient transfection

The cDNA encoding Gβγ sequester β-ARK-CT (gift of P. Voigt, Institute of Pharmacology, Charité-Medical Universiy, Campus Benjamin Franklin, Berlin, Germany) was transfected into subconfluent HEK-GHSR1a cells using Lipofectamine 2000 (Invitrogen; Carlsbad, CA, USA) following the manufacture's protocol. The β-ARK-CT incorporation was confirmed by means of intracellular calcium measurements in transfected cells before and after treatment as previously described [16].

### Immunoblotting analysis

Serum-starved cells were stimulated with ghrelin for the indicated time period at 37°C. The media was then aspirated and the cells were lysed in ice-cold RIPA buffer [Tris-HCl (pH 7.2), 50 mM; NaCl, 150 mM; EDTA, 1 mM; NP-40, 1% (v/v); Na-deoxycholate, 0.25% (w/v); protease inhibitor cocktail (Sigma; St. Louis, Mo, USA); phosphatase inhibitor cocktail (Sigma; St. Louis, Mo, USA)]. The solubilized lysates were transferred into centrifuge tubes and left at 4°C for 15 min, then pre-cleared by centrifuging at 13,000×*g* for 15 min. Protein concentration was evaluated with the QuantiPro™ BCA assay kit (Sigma; St. Louis, Mo, USA). Subsamples (same amount of protein) of each sample were separated on 10% SDS-polyacrylamide gels and transferred to nitrocellulose membranes. The blots were incubated with 5% non-fat dry milk in TBST [Tris-HCl (pH 8.0), 20 mM; NaCl, 150 mM; Tween-20, 0.1% (v/v); used for all incubation and washing steps] for 1 h. Next, blots were incubated for 1 h with the corresponding antibodies according to the manufacturer's instructions. The blots were subsequently incubated with the corresponding peroxidase-conjugated IgG antibody. After washing, signals were visualized using an enhanced chemiluminescence detection system (GE-Amersham; Buckinghamshire, UK).

### Immunoprecipitation

Serum-starved cells were stimulated with ghrelin for the indicated time period at 37°C and lysed in ice-cold non-denaturing NP-40 solubilization buffer [immunoprecipitation lysis buffer (ILB), Tris-HCl (pH 7.5), 20 mM; NaCl, 150 mM; EDTA, 1 mM; NP-40, 1% (v/v); protease inhibitor cocktail (Sigma, St. Louis, Mo, USA); phosphatase inhibitor cocktail (Sigma; St. Louis, Mo, USA)]. 500 µg of total protein was pre-washed with 20 µL of 50% protein A/G-agarose (Santa Cruz; Santa Cruz, CA, USA) for 30 min at 4°C, and then incubated with 1 µg of the corresponding antibody (overnight at 4°C) followed by addition of 40 µL of 50% protein A/G (2 h at 4°C). After washing two times with ILB, the pelleted beads were resuspended in Laemmli sample buffer. Proteins were analyzed by 10% SDS-polyacrylamide gels, followed by Western blotting.

### Small Interfering RNA (siRNA) Silencing of Gene Expression

Chemically synthesized double-stranded siRNA duplexes (with 3′ dTdT overhangs) were purchased from Santa Cruz Biothecnology for the following targets: β-arrestin 1 (5′-AAAGCCUUCUGCGCGGAGAAU-3′), β-arrestin 2 (5′-AAGGACCGCAAAGUGUUUGUG-3′, 5′-AAAGCCUUCUGCGCGGAGAAU-3′, 5′-AAGGACCGCAAAGUGUUUGUG-3′), Rictor (5′-CACUUCGAUUAGUCAGAAA-3′, 5′-CGCUUACUUUGCCUAACAA-3′, 5′-CCAACUGAGUGCAAUAUGU-3′), c-Src (5′-CUCGGCUCAUUGAAGACA-3′, 5′-UGACUGAGCUCACCACAAA-3′, 5′-CCUCAUCAUAGCAAUAACA-3′, 5′-GUAGAUUUCAGAUGACUAU-3′). A non-silencing RNA duplex was used as a control for all siRNA experiments. Cells were transfected with Lipofectamine 2000 (Invitrogen; Carlsbad, CA, USA), according to manufacturers' instructions. Silencing was quantified by immunoblotting. Only experiments with verified silencing were used.

### Data analysis

The results were expressed as the mean±S.E. Differences between means were evaluated by one-way analysis of variance (ANOVA). (*,#, *P*<0.05).

## Results

### Akt temporal pattern activation by ghrelin

**Figure 1:**
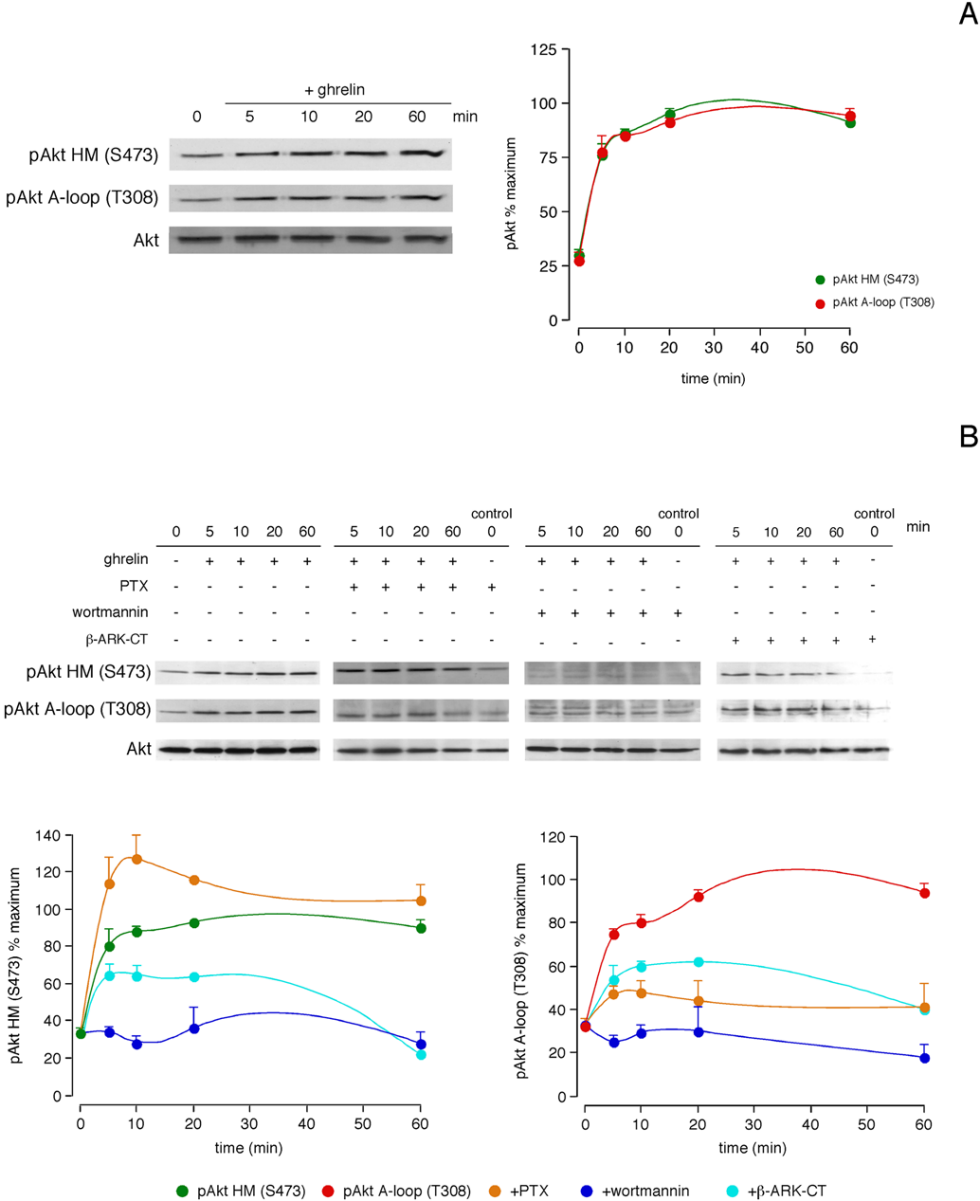
Role of G_i/o_ proteins, Gβγ dimers and PI3K in Akt phosphorylation in response to ghrelin. A, Time-course of the effect of ghrelin on Akt HM (S473) and A-loop (T308) phosphorylation. B, Ghrelin-induced Akt phosphorylation in the absence or presence of PTX (100 ng/mL, 12 h), PI3K inhibitor wortmannin (1 µM, 30 min) and βγ sequester β-ARK-CT. In A and B, serum-starved HEK-GHSR1a cells were treated with ghrelin (100 nM) at 37°C for the indicated times. Cells were lysed and analyzed by SDS-PAGE using specific antibodies. Akt phosphorylation was quantified by densitometry and expressed as a percentage of the maximal phosphorylation obtained for each residue (mean±S.E. of three independent experiments). Blots are representative of three independent experiments.

Figure 1A shows that Akt phosphorylation in both the activation loop within the kinase domain [A-loop (T308)] and the hydrophobic motif in the C-terminal region [HM (S473)] reached maximal levels within 20 min of ghrelin stimulation (100 nM), keeping the maximum by 60 min. The role of G_i/o_ protein was evaluated by means of pretreatment with pertussis toxin, which uncouples G_i/o_ protein from receptors (PTX; 100 ng/mL, 12 h). As shown in Figure 1B, PTX reduced the ghrelin-induced phosphorylation of the Akt A-loop (T308). Surprisingly, PTX pretreatment showed an increase of Akt HM (S473) phosphorylation after ghrelin stimulation. Transfection with Gβγ sequesters, β-ARK-CT, decreased the effect of ghrelin on Akt phosphorylation at both residues, indicating the involvement of the βγ-subunit of G proteins (Fig. 1B). Pretreatment with the PI3K inhibitor wortmannin (1 µM, 30 min) decreased the ghrelin-induced Akt phosphorylation at both residues (Fig. 1B).

### c-Src tyrosine kinase regulates Akt activity

**Figure 2:**
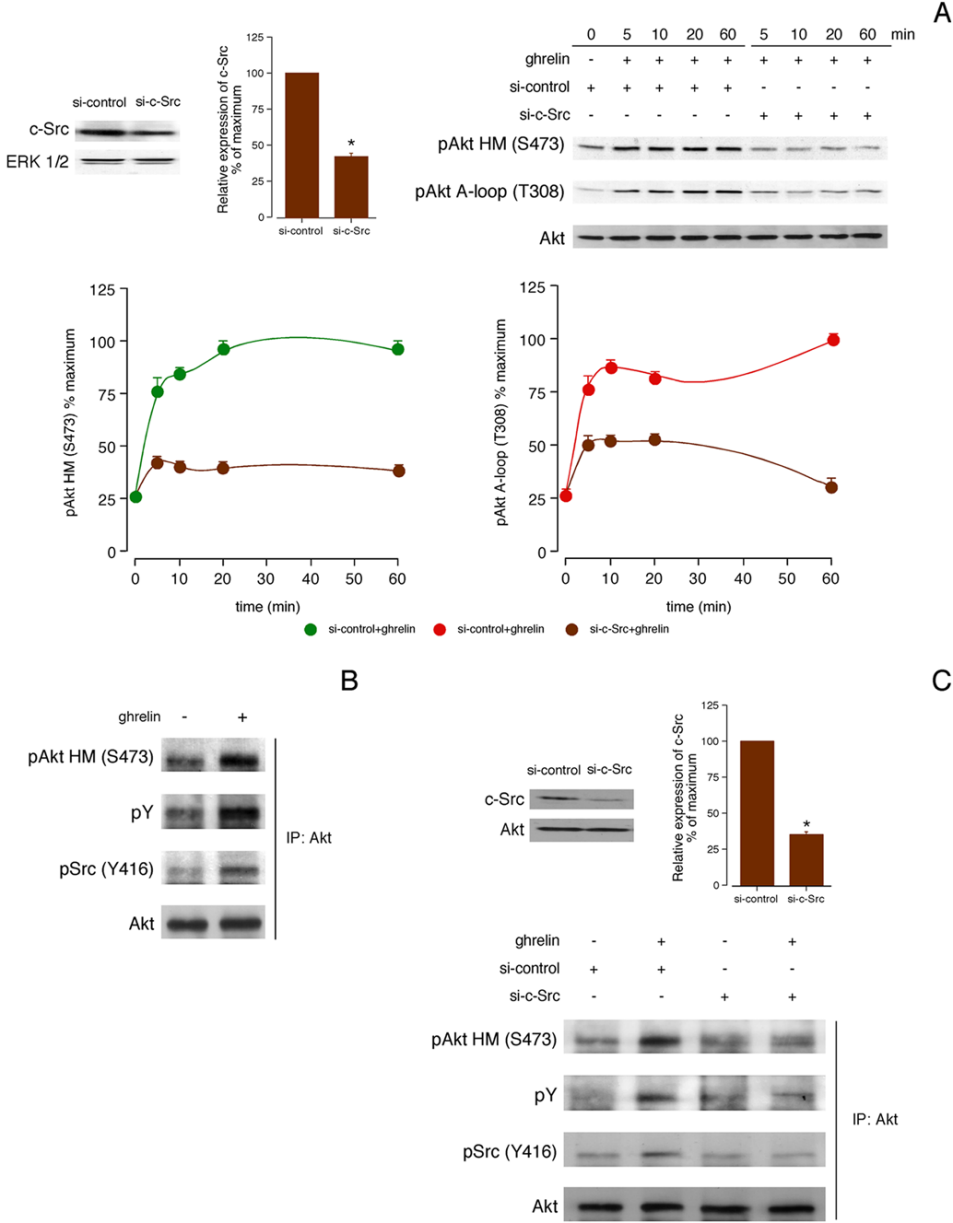
c-Src is required for phosphorylation of Akt in response to ghrelin. A, Effect of siRNA depletion of c-Src on ghrelin-induced Akt phosphorylation. HEK-GHSR1a cells transfected with c-Src siRNA were serum-starved for 12 h and then stimulated with ghrelin (100 nM) at 37°C. After stimulation, equal amounts of protein in each sample were used to assess the expression of c-Src (left panel) and Akt phosphorylation (right panel) by western blotting. Expression of c-Src was quantified by densitometry and expressed as percentages of the level of c-Src in control siRNA-transfected cells (mean±S.E.). Akt phosphorylation was quantified by densitometry and expressed as a percentage of the maximal phosphorylation at HM (S473) and A-loop (T308) after ghrelin addition to control siRNA-transfected cells (mean±S.E.). B, Effect of ghrelin on Y phosphorylation of Akt and interaction between Akt and c-Src. Cells were incubated with ghrelin (100 nM, 5 min) at 37°C, lysed and immunoprecipitated (IP) with antibodies to Akt, and then analyzed by western blotting with pAkt HM(S473), pY, pSrc (Y416) antibodies. C, Effect of ghrelin on Y phosphorylation of Akt in the presence of c-Src siRNA. HEK-GHSR1a cells transfected with c-Src siRNA were serum-starved for 12 h and then stimulated with ghrelin (100 nM, 5 min) at 37°C. Equal amounts of protein in each sample were used to assess the expression of c-Src [upper panel; values shown (mean±S.E.) are percentages of the level of c-Src in control siRNA-transfected cells]. Cells were lysed and immunoprecipitated (IP) with antibodies to Akt, and then analyzed by western blotting with with pAkt HM(S473), pY, pSrc (Y416) antibodies. Immunoblots are representative of three independent experiments.

Akt phosphorylation was strongly inhibited at both residues by the selective Src inhibitor PP2 (5 µM, 30 min) for all time tested. This inhibition was specific, since pretreatment with PP3 (5 µM, 30 min), a negative control for PP2, had no effect on the ghrelin-induced Akt phosphorylation (data not shown). As shown in Figure 2A, siRNA experiments targeting c-Src reduced its expression by 57±2% (Fig. 2A). In the presence of a non-targeting control siRNA, ghrelin-activated Akt phosphorylation was identical to that observed without any transfection. c-Src siRNA decreased ghrelin-activated Akt phosphorylation with respect to siRNA control for all time tested [54±3% at HM(S473); and, 45±6% at A-loop(T308)]. Immunoprecipitation assays of Akt showed an increase of Y phosphorylation of this kinase in ghrelin-treated cells (100 nM, 5 min) in comparison with untreated cells (Fig. 2B). Furthermore, the activated form of c-Src, pSrc (Y416), co-immunoprecipitated with Akt, which demonstrates a direct interaction between both kinases. Immunoprecipitation assays of Akt in the presence of c-Src siRNA (66±2% reduction in c-Src expression) clearly decreased Y phosphorylation of this kinase in ghrelin-treated cells (100 nM, 5 min) in comparison with non-targeting control siRNA cells (Fig. 2C). c-Src siRNA decreased ghrelin-activated Akt phosphorylation and coimmunoprecipitation of pSrc (Y416) with respect to siRNA control.

### 3-phosphoinositide-dependent protein kinase 1 (PDK1) and mammalian rapamycin-insensitive complex 2 (mTORC2) mediate Akt phosphorylation activated by ghrelin

**Figure 3:**
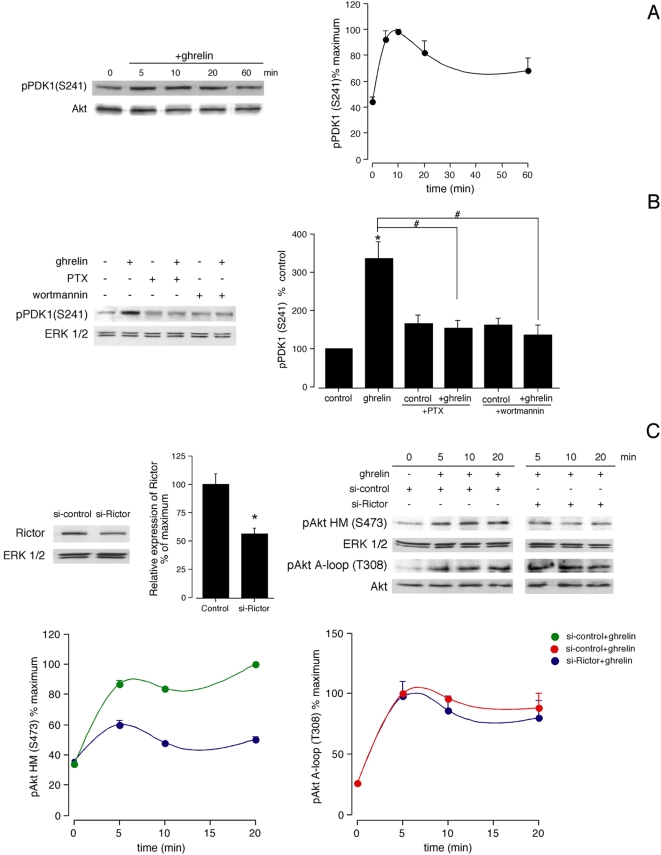
PDK1 and mTORC2 regulate Akt phosphorylation activated by ghrelin. A, Time-course of the effect of ghrelin (100 nM) on phosphorylation of PDK1. Cell extracts were analyzed by SDS-PAGE using specific antibody against pPDK1 (S241) and Akt. PDK1 phosphorylation was quantified by densitometry and expressed as a percentage of the maximal phosphorylation (mean±S.E of three independent experiments). B, PDK1 phosphorylation induced by ghrelin (100 nM, 5 min) in the absence or presence of PTX (100 ng/mL, 12 h) and the PI3K inhibitor wortmannin (1 µM, 30 min). PDK1 phosphorylation was quantified by densitometry and expressed as the percentage of the basal phosphorylation obtained in control cells (means±S.E.). C, Effects of siRNA depletion of Rictor on ghrelin-induced Akt phosphorylation. HEK-GHSR1a cells transfected with Rictor siRNA were stimulated with ghrelin (100 nM) at 37°C. After stimulation, cell extracts were prepared as described in Experimental Procedures. Equal amounts of protein in each sample were used to assess the expression of Rictor and Akt phosphorylation by Western blotting. Expression of Rictor was quantified by densitometry. Shown values are percentages of the level of Rictor in control siRNA-transfected cells. Akt phosphorylation was quantified by densitometry and expressed as a percentage of the maximal phosphorylation of Akt HM (S473) and A-loop (T308) after ghrelin stimulation to control siRNA-transfected cells (mean±S.E.). In A, B and C, blots are representative of three independent experiments.

Figure 3A shows that pPDK1 (S241) reached maximal levels within 5–10 min of ghrelin stimulation (100 nM), decreasing to ∼50% of the maximum by 60 min after stimulation. This increase was parallel to the phosphorylation of Akt A-loop (T308). Pretreatment of cells with PTX (100 ng/mL, 12 h) had a significant inhibitory effect on ghrelin-induced pPDK1 (S241) (100 nM, 5 min). Inhibition of PI3K by wortmannin pretreatment (1 µM, 30 min) inhibited the ghrelin-induced pPDK1 (S241) (Fig. 3B). On the other hand, working in the presence of Rictor siRNA (50±2% reduction in Rictor expression; Fig. 3C), Akt HM (S473) phosphorylation was reduced by 50±4%, whereas A-loop (T308) phosphorylation was not affected (Fig. 3C).

### Regulation of Akt phosphorylation by β-arrestins

**Figure 4:**
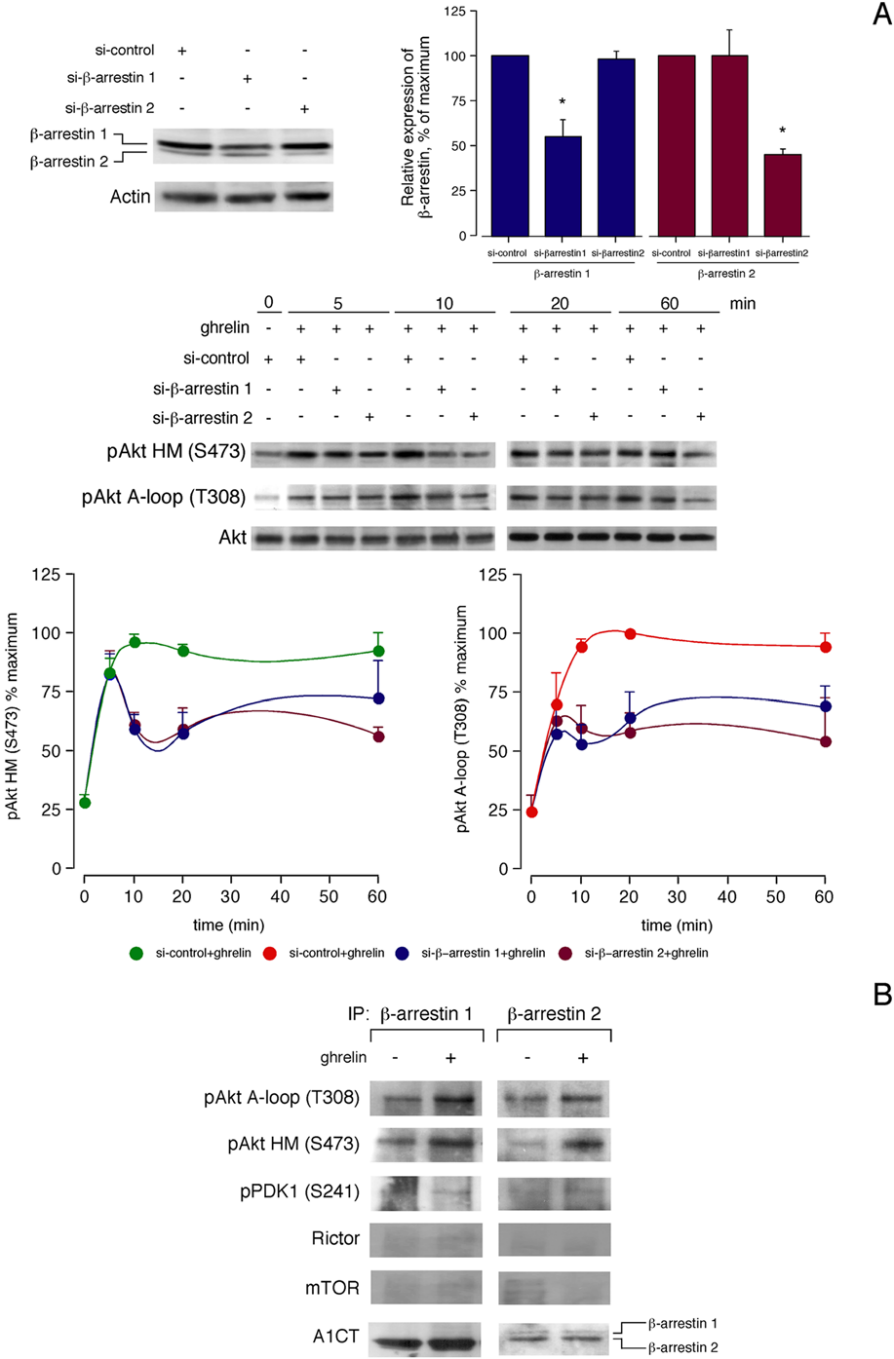
β-arrestins are required for the modulation of Akt phosphorylation in response to ghrelin. A, Effect of siRNA depletion of β-arrestin 1 or β-arrestin 2 on ghrelin-induced Akt phosphorylation. HEK-GHSR1a cells transfected with β-arrestin 1 or β-arrestin 2 siRNA were serum-starved for 12 h and then stimulated with ghrelin (100 nM) at 37°C. After stimulation, equal amounts of protein in each sample were used to assess the expression of β-arrestin 1 or β-arrestin 2 (upper panel) and Akt phosphorylation (lower panel) by Western blotting. Expression of β-arrestin 1 or β-arrestin 2 was quantified by densitometry. Values shown are percentages of the level of β-arrestins in control siRNA-transfected cells. Akt phosphorylation was quantified by densitometry and expressed as a percentage of the maximal phosphorylation at HM (S473) and A-loop (T308) after ghrelin addition to control siRNA-transfected cells (mean±S.E.). B, Effect of ghrelin on the assembly of complexes containing β-arrestins 1 and 2 and pAkt. Cells were incubated with ghrelin (100 nM, 10 min) at 37°C, lysed and immunoprecipitated (IP) with antibodies to β-arrestin 1 (left panel) or β-arrestin 2 (right panel), and then analyzed by western blotting with pAkt[HM(S473), A-loop(T308), pPDK1 (S241)], Rictor, mTOR, and β-arrestin (A1CT) antibodies. In A and B, western blots are representative of three independent experiments.

siRNA experiments targeting β-arrestin 1 or β-arrestin 2 reduced their expression by 45±2% and 55±4%, respectively (Fig. 4A). In the presence of a non-targeting control siRNA, ghrelin-activated Akt phosphorylation was identical to that observed without any transfection (data not shown). β-arrestin 1 and β-arrestin 2 siRNA led to rapid Akt phosphorylation, which decreased after 10 min of ghrelin stimulation with respect to siRNA control [41±2% and 39±3% at HM(S473) for β-arrestin 1 and β-arrestin 2 siRNA, respectively; and, 47±5% and 40±4% at A-loop(T308), for β-arrestin 1 and β-arrestin 2 siRNA, respectively (Fig. 4A)]. This inhibitory effect stayed on both residues for at least 60 min. Immunoprecipitation of ghrelin-treated cells (100 nM, 10 min) with antibodies for β-arrestin 1 or β-arrestin 2 coprecipitated pAkt (T308, S473), while failed to coprecipitate pPDK1 (S241), Rictor and mTOR (Fig. 4B). The β-arrestin-dependent Akt phosphorylation was dependent on Src as no immunoprecipitation of pAkt was obtained with antibodies to β-arrestin 1 or β-arrestin 2 in ghrelin stimulated cells (100 nM, 10 min) after pretreatment with PP2 (5 µM, 30 min). This inhibition was specific, since pretreatment with PP3 (5 µM, 30 min) had no effect on the β-arrestin-dependent Akt phosphorylation induced by the agonist (data not shown).

### Regulation of Akt phosphorylation by Src and β-arrestins in 3T3-L1 preadipocyte cells

**Figure 5:**
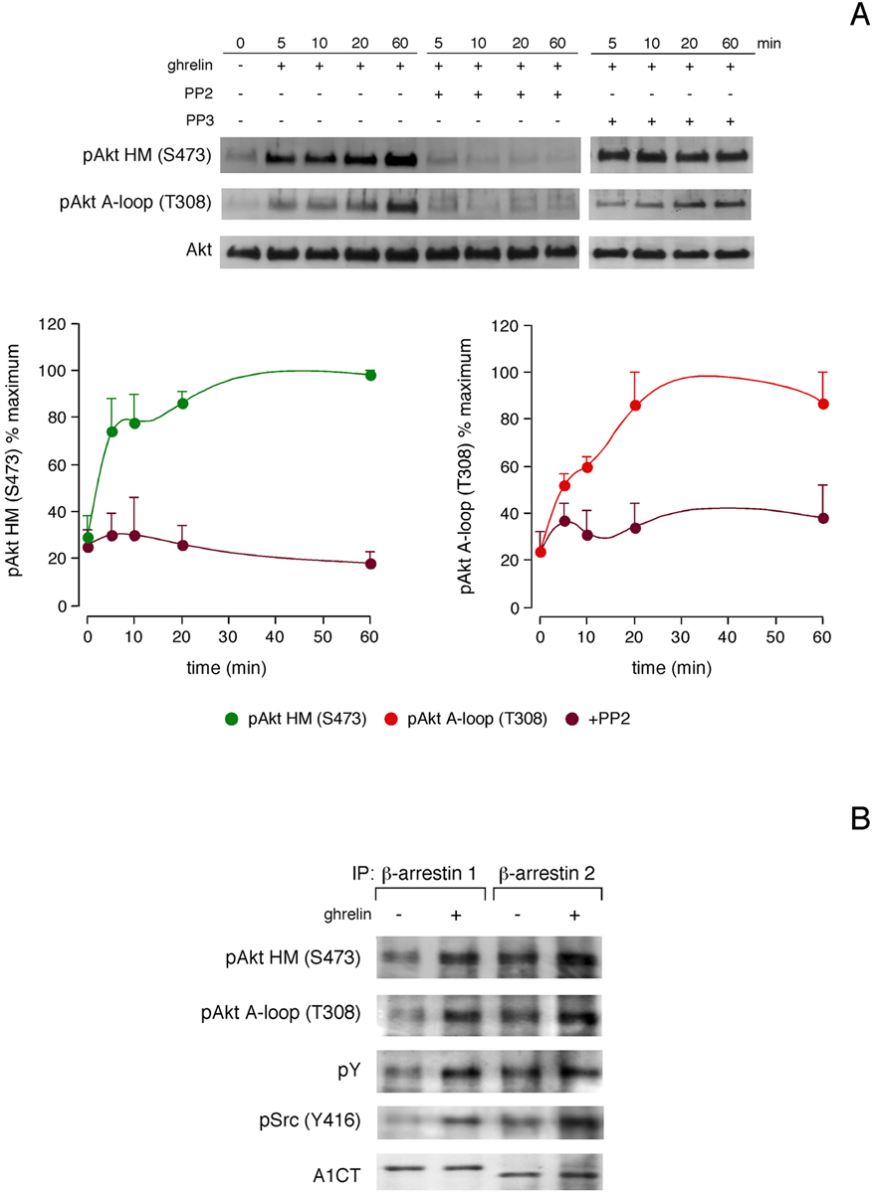
Src and β-arrestins regulate Akt phosphorylation in response to ghrelin in 3T3-L1 preadipocyte cells. A, Effects of the Src inhibitor PP2 and its negative control, PP3, on the ghrelin-induced Akt activation in 3T3-L1 preadipocyte cells. Serum-starved cells were pretreated with PP2 (5 µM, 30 min) or PP3 (5 µM, 30 min) before ghrelin stimulation (100 nM) for the indicated time periods. Akt phosphorylation was quantified by densitometry and expressed as a percentage of the maximal phosphorylation at HM (S473) and A-loop (T308) (mean±S.E of three independent experiments). B, Effect of ghrelin on the assembly of complexes containing β-arrestins 1 and 2 and pAkt. Preadipocyte 3T3-L1 cells were incubated with ghrelin (100 nM, 10 min) at 37°C, lysed and immunoprecipitated (IP) with antibodies to β-arrestins 1 and 2, and then analyzed by western blotting with pAkt HM (S473), pAkt A-loop (T308), pY, pSrc (Y416) antibodies. For A and B, western blots are representative of three independent experiments.

As shown in Figure 5A, both Akt A-loop (T308) and HM (S473) phosphorylation reached maximal levels within 20–60 min of ghrelin addition (100 nM). The Akt phosphorylation was inhibited at both residues by pretreatment with PP2 (5 µM, 30 min) (Fig. 5A). Immunoprecipitation of ghrelin-treated cells (100 nM, 10 min) with antibodies for β -arrestin 1 or β-arrestin 2 coprecipitated full-activated Akt showing an increase on tyrosine phosphorylation compared to untreated cells (Fig. 5B). Furthermore, the activated form of Src, pSrc (Y416), co-immunoprecipitated with β-arrestin 1 or β-arrestin 2 (Fig. 5B).

## Discussion

The present study offers three major findings related to the activation of Akt in response to ghrelin. First, Akt is phosphorylated by the interplay of two G_i/o_-protein-dependent signaling pathways. One pathway is Gβγ-dependent and involves the activation of PI3K. The second pathway is mediated by the β-arrestins 1 and 2, and requires the entry of the receptor into a multiprotein complex. Second, PDK1 and mTORC2 are essential for A-loop and HM phosphorylation of Akt, respectively. Third, c-Src plays an essential role in the activation of Akt mediated by ghrelin. Thus, ghrelin activates Akt pools that differ in their temporal and spatial distributions, suggesting to have different physiological targets.

The protein kinase Akt has emerged as a pivotal signaling node that regulates the control of cell proliferation, nutrient uptake and metabolism in a cell-type-specific manner through a variety of down-stream targets [7]. From the results presented so far, ghrelin activates Akt by two distinct phosphorylation events, both of which depend on PI3K. GHS-R1a activates PI3K through G_i/o_ protein to regulate several downstream signaling pathways through the generation of the lipid second messenger phosphatidylinositol-3,4,5-triphosphate [PtdIns(3,4,5)P_3_] [6]. Since expression of Gβγ sequestrant inhibited both Akt HM (S473) and A-loop (T308) phosphorylation, we suggest that Gβγ dimers from G_i/o_ are the upstream signal for PI3K activation [17]. PtdIns(3,4,5)P_3_ allows membrane translocation of proteins containing PH domain such as PDK1 and Akt. Then, PDK1 is autophosphorylated at Ser241 leading to its own activation and consequently phosphorylates Akt A-loop (T308) [18], [19]. Finally, the full activation of Akt involves the phosphorylation of HM (S473). Several kinases, designated as PDK2 kinases, have been proposed to phosphorylate the Akt HM (S473) kinase; from those at least 10 of them may function as PDK2, including integrin-linked kinase (ILK), protein kinase Cα (PKCα), double-stranded DNA-dependent protein kinase (DNA-PK), ataxia telangengiectasia mutated (ATM) gene product, and the mTORC2 [20]. From all the proposed candidates, evidence supports mTORC2 as the most compelling. mTORC2 contains Rictor, mLST8, mSin1 variants and the mTOR kinase [21]. Our study shows that ablating mTORC2 function by siRNA targeting Rictor impaired ghrelin-stimulated HM (S473) phosphorylation of Akt, but not at the A-loop (T308). This result identifies the hypothetical ghrelin-activated PDK2 as mTORC2. The fact that the Akt A-loop (T308) phosphorylation is unaffected by loss of HM (S473) phosphorylation in mTORC2-deficient cells, supports that the phosphorylation of HM (S473) is not critical for that of A-loop (T308). Thus, the PDK1-mediated Akt A-loop (T308) phosphorylation is not dependent on prior HM (S473) phosphorylation by mTORC2 [22]–[25]. Interestingly, PTX treatment selectively enhanced earliest time points of Akt HM (S473) phosphorylation, consistent with a regulatory role of Gα_i/o_ protein. This opens the possibility that Gα_i/o_ regulates the activation of the recently discovered PH domain leucine-rich repeat phosphatase (PHLPP1 and PHLPP2), phosphatases that selectively dephosphorylate the HM of Akt, resulting in decreased kinase activity [26], [27]. These results support the model by which both Akt and PDK1 interact by colocalization at the plasma membrane, which is further phosphorylated by mTORC2 [21]. Despite being a key element in the Akt activation, the way mTORC2 is activated remains unknown. Based on the PH-like domain of mSIN1, mTORC2 and Akt might interact as consequence of colocalization at plasma membrane when PI3K is activated [28]. This does not exclude the possibility that other upstream signals regulate phosphorylation/activation of the mTORC2 in response to different growth factors. Indeed mSIN1 contains a Ras-binding domain raising the possibility that Ras regulates mTORC2 [28], [29].

c-Src kinase is involved in the MAPK activation in response to ghrelin. Ghrelin induces a first “wave” of c-Src activation via G protein-dependent signaling that it is followed by a second wave of c-Src activation via β-arrestins-mediated signaling [6]. In the present work, c-Src acts upstream of Akt, as c-Src depletion using siRNAs reduced the ghrelin-induced Akt phosphorylation at both A-loop (T308) and HM (S473). It was reported that, in addition to phosphorylation of A-loop (T308) and HM (S473), Y phosphorylation is also essential for Akt activation [30], [31]. Our data indicate that ghrelin activates Y phosphorylation of Akt, although the detailed mechanism of this regulation remains to be elucidated. Additionally, coimmunoprecipitation assays showed that c-Src interacts with Akt in response to ghrelin. Because c-Src contains an SH3 domain, it was proposed that Src interacts with the PXXP motif of Akt [32]. Our data are consistent with this previously proposed model in which Akt translocates to the plasma membrane through the binding of its PH domain to PtdIns(3,4,5)P_3_ generated by PI3K. This allows the interaction of membrane bound Src and Akt through its PXXP motif in the C-terminal regulatory region and the SH3 domain of Src. Then Src phosphorylates Akt at Y residue(s), which triggers Akt A-loop (T308) and HM (S473) phosphorylation by PDK1 and mTORC2 [32]. These data support the idea that Src operates as a “switch” in concert with PI3K for activation of Akt in response to ghrelin.

The existence of a pathway involving receptor endocytosis is supported by the fact that β-arrestin depletion using siRNAs reduced the magnitude of Akt activation by ghrelin. Our coimmunoprecipitation assays indicate that the β-arrestins function as adaptors recruiting Akt to the ghrelin-occupied receptor through the formation of β-arrestin complexes. The mechanism of activation of Akt-associated to β-arrestins is not completely delineated. From the data presented so far, c-Src is essential for Akt activation even when is associated to β-arrestins. Given that c-Src is recruited to GHS-R1a upon ghrelin stimulation [6], it would be possible that Akt interacts with Src associated to GHSR1a-β-arrestin complex as consequence of colocalization at plasma membrane when PI3K is activated in response to ghrelin. This Akt-c-Src association is presumed to initiate Akt phosphorylation by allowing Y phosphorylation. Furthermore, β-arrestin-scaffolded complex presumably places the different components of the Akt cascade in close proximity to each other and thus ensuring substrate specificity. This is supported by the fact that neither PDK1 nor mTORC2 coimmunoprecipitated with β-arrestins. Interestingly, both β-arrestin 1 and β-arrestin 2 are required to promote the activation of Akt. The requirement of both proteins might be indicative of a need to form heterodimers to activate the β-arrestin-dependent signaling pathway as it was described for MAPK activation [6]. However, this cannot be generalized, since in other systems β-arrestins show opposite effects. For example, in the case of the D_2_-class receptors, β-arrestin 2 facilitates the dephosphorylation of Akt by phosphatase 2 (PP2A) in response to dopamine [33], [34]. In addition, prostaglandin E_2_ [35], IGF-1 [36], α-thrombin [37] and β1-adrenergic receptors [38], [39] activate Akt pathway through β-arrestin 1 dependent mechanisms. It seems that each receptor determine the β-arrestin-associated functions on basis of the receptor-associated isoforms.

**Figure 6:**
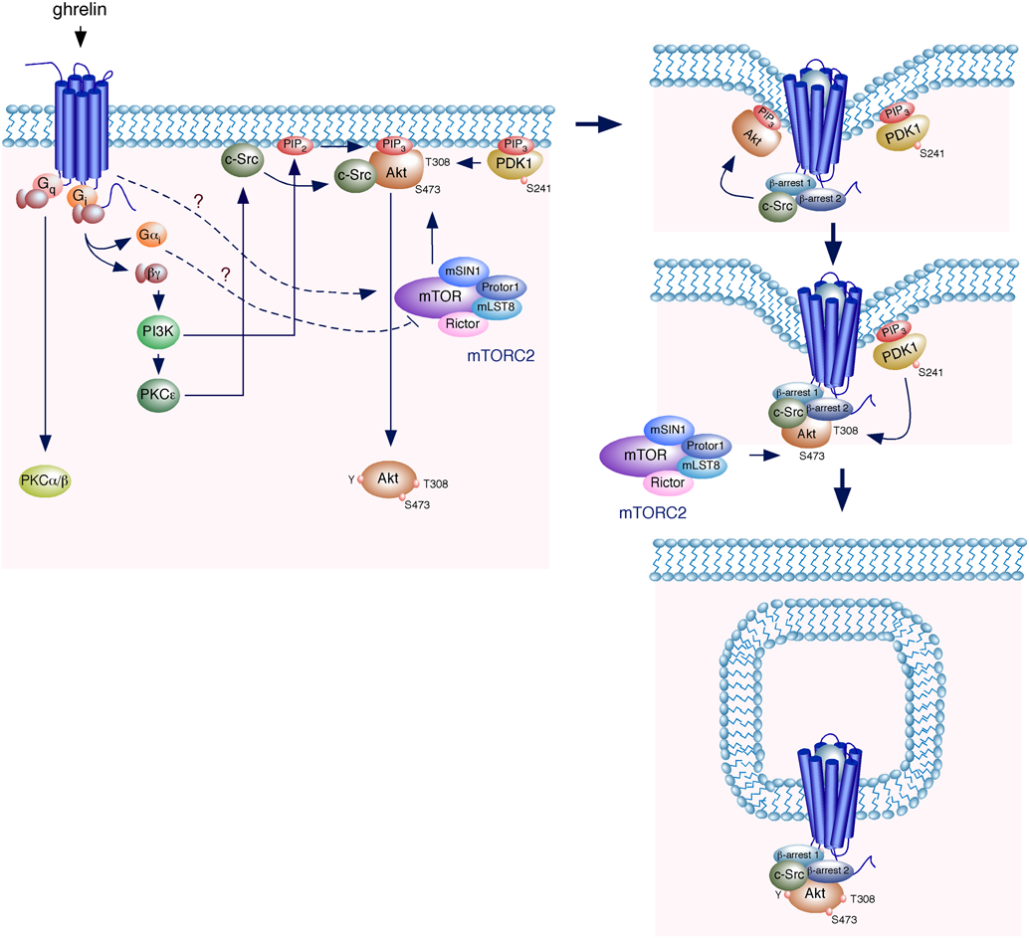
Model of upstream activation of Akt by ghrelin. Binding of ghrelin to GHS-R1a activates the generation of the second messenger PtdIns(3,4,5)P_3_ (PIP3) by PI3K activation through G_i/o_-protein dependent signaling pathway. Akt translocates to the plasma membrane by binding to PIP3 where it is phosphorylated at Y by the membrane bound c-Src. This phosphorylation (Y) is followed by phosphorylation of Akt A-loop (T308) and HM (S473) by PDK1 and mTORC2 respectively. Once the receptor is activated, a second signaling pathway is mediated by β-arrestins 1 and 2, involving the recruitment of GHS-R1a, c-Src and Akt into an β-arrestin-scaffolded complex.

It has been shown that ghrelin regulates the Akt activity through the GHS-R1a in a variety of cellular systems [8]–[15]. Particularly, the mitogenic effect of ghrelin in 3T3-L1 preadipocytes is mediated by the PI3K/Akt and MAPK pathways, via a G_i_-protein [14]. Accordingly to our results in HEK-GHSR1a cells, co-immunoprecipitation assays performed in 3T3-L1 cells indicate that β-arrestins recruit Akt, leading to its activation. c-Src acts upstream of Akt in these cells, as PP2 abolished the ghrelin-induced Akt phosphorylation at both residues. Furthermore, co-immunoprecipitation assays showed that c-Src interacts with Akt in response to ghrelin allowing the phosphorylation of Akt at Y residue(s). Therefore, our data are consistent with a model in which Akt translocates to the plasma membrane through the binding of its PH domain to the second messenger PtdIns(3,4,5)P_3_ generated by PI3K which is activated through G_i/o_-protein dependent signaling pathway. Akt is phosphorylated at Y by the membrane bound Src via the interaction between its C-terminal proline-rich motif and the SH3 domain of Src. This Y phosphorylation is followed by phosphorylation of Akt A-loop (T308) and HM (S473) by PDK1 and mTORC2 respectively. Once the receptor is activated, a second signaling pathway is mediated by β-arrestins 1 and 2 involving the recruitment of GHS-R1a, c-Src and Akt into an β-arrestin-scaffolded complex (Fig. 6). Thus c-Src functions as a switch that initiates the Akt pathway associated to both the G_i/o_-protein dependent pathway and β-arrestin-scaffolded complex.
